# Effects of Water Availability on the Relationships Between Hydraulic and Economic Traits in the *Quercus wutaishanica* Forests

**DOI:** 10.3389/fpls.2022.902509

**Published:** 2022-05-26

**Authors:** Yuhan Zhang, Jiale Zhao, Jinshi Xu, Yongfu Chai, Peiliang Liu, Jiaxin Quan, Xipin Wu, Cunxia Li, Ming Yue

**Affiliations:** ^1^Key Laboratory of Resource Biology and Biotechnology in Western China, Northwest University, Xi'an, China; ^2^Xi'an Botanical Garden of Shaanxi Province/Institute of Botany of Shaanxi Province, Xi'an, China

**Keywords:** functional trait, congeneric species, trade-off, species distribution, temperate forest

## Abstract

Water availability is a key environmental factor affecting plant species distribution, and the relationships between hydraulic and economic traits are important for understanding the species’ distribution patterns. However, in the same community type but within different soil water availabilities, the relationships in congeneric species remain ambiguous. In northwest China, *Quercus wutaishanica* forests in the Qinling Mountains (QM, humid region) and Loess Plateau (LP, drought region) have different species composition owing to contrasting soil water availability, but with common species occurring in two regions. We analyzed eight hydraulic traits [stomatal density (SD), vein density (VD), wood specific gravity (WSG_branch_), lower leaf area: sapwood area (Al: As), stomatal length (SL), turgor loss point (Ψ_Tlp_), maximum vessel diameter (Vd_max_) and height (Height)] and five economic traits [leaf dry matter content (LDMC), leaf tissue density (TD), leaf dry mass per area (LMA), Leaf thickness (LT) and maximum net photosynthetic rate (P_max_)] of congeneric species (including common species and endemic species) in *Q. wutaishanica* forests of QM and LP. We explored whether the congeneric species have different economic and hydraulic traits across regions. And whether the relationship between hydraulic and economic traits was determined by soil water availability, and whether it was related to species distribution and congeneric endemic species composition of the same community. We found that LP species tended to have higher SD, VD, WSG_branch_, Al: As, SL, Ψ_Tlp_ and Vd_max_ than QM species. There was a significant trade-off between hydraulic efficiency and safety across congeneric species. Also, the relationships between hydraulic and economic traits were closer in LP than in QM. These results suggested that relationships between hydraulic and economic traits, hydraulic efficiency and safety played the role in constraining species distribution across regions. Interestingly, some relationships between traits changed (from significant correlation to non-correlation) in common species across two regions (from LP to QM), but not in endemic species. The change of these seven pairs of relationships might be a reason for common species’ wide occurrence in the two *Q. wutaishanica* forests with different soil water availability. In drought or humid conditions, congeneric species developed different types of adaptation mechanisms. The study helps to understand the environmental adaptive strategies of plant species, and the results improve our understanding of the role of both hydraulic and economic traits during community assembly.

## Introduction

Water availability is an essential resource for plants survival, growth and distribution (Toledo et al., 2012; Poorter et al., 2017; Ramírez-Valiente and Cavender-Bares, 2017; Ali et al., 2018; Granato-Souza et al., 2018; Rodríguez-Ramírez et al., 2019). The long-term adaptation of plants to different water conditions will originate different water regulation strategies (Aguilar-Romero et al., 2017; Luo et al., 2017; Liu et al., 2021a) and functional traits (Rita et al., 2016). Different strategies of plants in response to the environment, such as water transport, mechanical support, and defense strategies, combine to determine the survival and distribution of plants (Bucci et al., 2012; Reich, 2014). Therefore, the relationships between functional traits and species distribution in multiple dimensions of traits should be studied (Laughlin, 2014; Liu et al., 2021c). Previous studies have focused on economic traits (Zheng and Shangguan, 2007a,b,c; Tanaka-Oda et al., 2010; Suter and Edwards, 2013; Chai et al., 2015; Zhang et al., 2017; Han et al., 2020; Ji et al., 2020), which reflect trade-offs between acquisition and investment of resources (Wright et al., 2004). Economic traits are related to CO_2_ and water exchange and light capture, such as maximum net photosynthetic rate (P_max_; Li et al., 2015). However, it is of great ecological significance to explore the water limitation in vegetative growth through hydraulic traits, which could reflect these resource allocation strategies as well (von Arx et al., 2012). Economic and hydraulic traits reveal different response levels of plants to environmental changes (Yin et al., 2018; Zhao et al., 2021). Hydraulic traits are related to water transport and loss, which affect plants’ water transport efficiency and gas exchange and thus affect economic traits (i.e., the rate of photosynthesis; Liu et al., 2021b). Hydraulic traits play a key role in limiting species’ growth, competition and distribution (Meinzer et al., 1999; Brodribb et al., 2005; Tomasella et al., 2008; Villagra et al., 2013; Cosme et al., 2017), which are more directly reflection of water availability.

Species may exhibit a combination of hydraulic traits under different soil water availability, which can be related to the ability to tolerate drought or wetness (von Arx et al., 2012). The combination of divergent species hydraulic traits suggests a trade-off between hydraulic efficiency and safety (Tyree et al., 1994; Cosme et al., 2017). Normally, the combination follows biophysical rules, that is, hydraulic efficiency (i.e., large and grouped vascular bundles) and hydraulic safety (i.e., narrow and isolated vascular bundles) cannot coexist (Litvak et al., 2012; Liu et al., 2020b). In low soil water availability, plants must take a conservative strategy to ensure hydraulic safety by investing in traits that can improve water-resistance, avoid cavitation, minimize the risk of embolism (i.e., to conduct water with narrow vessels; Zhao et al., 2021). By contrast, plants must invest in traits that confer high hydraulic efficiency in humid regions (i.e., to conduct water with wide vessels) to reduce the resistance of water flow and to increase conductivity (Cosme et al., 2017). A recent integrated analysis, however, has shown only weak support for the trade-off between hydraulic efficiency and safety (Gleason et al., 2016b; Schuldt et al., 2016; Zhu et al., 2017; Santiago et al., 2018), causing some controversy (Bittencourt et al., 2016; Brodersen, 2016; Gleason et al., 2016a). The combinations of hydraulic traits for species under different soil water availability remain unclear. Cosme et al. (2017) argued that there was hydraulic safety vs. efficiency trade-off in different water conditions, which affects species co-occurrence. The results were obtained in congeneric endemic species mostly restricted to plateaus (drought) or valleys (humid). However, Zhu et al. (2017) showed the opposite results: in two forests with different water conditions, the relationships of congeneric common species remained decoupled, which have high hydraulic efficiency and safety at the same time. Therefore, the relationships between hydraulic efficiency and safety should be tested closely. Both congeneric endemic species and common species should be considered.

Previous studies found that hydraulic traits and economic traits were decoupled (Li et al., 2015; Zhang et al., 2015; Liu et al., 2020a). The decoupled relationships brought greater freedom for more combinations of leaf traits to adapt to different environments (Li et al., 2015). But Yin et al. (2018) indicated hydraulic and economic traits may be coupled in semi-arid regions and the relationships depend on water availability. Similar results were also found in Li et al. (2018) and Males and Griffiths (2018). However, the physiological effects of the different relationships are unknown. In addition, Zhu et al. (2017) showed that common species can be found commonly in two regions owing to escaping hydraulic trade-off. Whether the relationships between hydraulic and economic traits would affect plant species distribution is still unclear. Exploring the relationships between hydraulic and economic traits under different water availability may help reveal how water availability shapes plant communities (Kraft et al., 2008).

Since the Middle Pleistocene, *Quercus wutaishanica* forest has become the dominant and stable community in specific areas of Qinling Mountains (QM, humid region) and Loess Plateau (LP, drought region; Zhu, 1982). *Quercus wutaishanica* forests in QM and LP have different species composition owing to different soil water availability (Yue, 1998; Zhao et al., 2021), but with some species that can be found commonly in two forests. There are congeneric endemic species with complementary distributions (For each genus, a species mostly restricted to LP and a species mostly restricted to QM) and congeneric common species distributed in two regions. It is necessary to explore the causes of the distribution patterns through economic and hydraulic traits.

In the present study, we investigated the relationships between hydraulic and economic traits of congeneric species (common and endemic species) in *Q. wutaishanica* forests which are disjunctively distributed in QM and LP. We aim to understand why species composition differs within the same community type, revealing the physiological and ecological mechanisms of species niche differentiation. We hypothesized that in soils with different water availability, (1) there was a trade-off between hydraulic safety and efficiency across congeneric species (common species and endemic species), which might affect species co-occurrence at the regional scale. (2) The relationships between hydraulic and economic traits were closer in drought region than in humid region, which might be a type of adaptation mechanism. (3) Common species might change the relationships between hydraulic and economic traits, which might provide an explanation for their common occurrence in the two *Q. wutaishanica* forests.

## Materials and Methods

### Study Sites and Plant Materials

The present study was performed in natural *Q. wutaishanica* forests in Shaanxi province, northwest China. Based on our previous work, both Ziwuling region (35°41′–35°44′ N, 109°00′–109°02′ E) on the middle section of LP and Taibaishan Nature Reserve (33°84′–33°86′ N, 108°82′–108°87′ E) in the north slope of QM were selected as the sampling sites. The climate of *Q. wutaishanica* forests in LP is semiarid, temperate, continental monsoon, with a mean annual temperature of 9°C–11°C (Chai et al., 2014). The mean annual precipitation is approximately 560–650 mm. The soil type is cinnamon soil (Liang et al., 2010). The northern boundary of its distribution is determined by water (Meng et al., 2011; Yin et al., 2018). The climate of *Q. wutaishanica* forests in QM is temperate monsoon, with a mean annual temperature of 6.5°C, and its upper limit of distribution is determined by temperature (Zhu et al., 1982). The mean annual precipitation is approximately 900–1,000 mm, which is relatively humid. The soil type is brown soil (Zhu et al., 1982). There are also significant differences in soil water content under such different climatic conditions (Supplementary Figure S1; Zhao et al., 2021).

**Figure 1:**
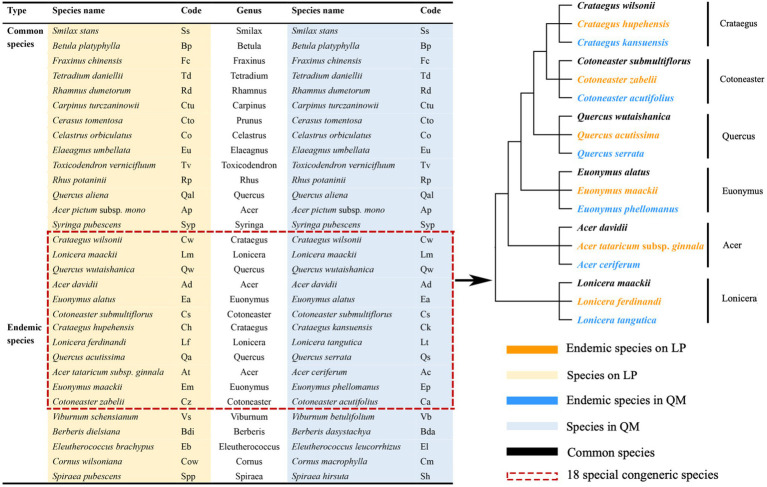
List of 31 pairs of congeneric species (including 20 pairs of congeneric common species and 11 pairs of congeneric endemic species) in *Quercus wutaishanica* forests across two regions and evolutionary relationship for 18 special congeneric species in six genera selected for this study.

This research was surveyed in July 2019, three plots (50 × 50 m) were established in the *Q. wutaishanica* forests of LP (35°37′–35°49′N, 109°00′–109°11′E; at 1100–1112 m) and QM (33°84′–34°41′N, 107°45′–108°83′E; 1900–1958 m), and the geographic information is the same as our previous work (Table 1 in Zhao et al., 2021). The total number of common species in the two communities is less than 15% (Zhao and Yue, 1996; Yue, 1998). The species selected in this study are the most abundant species of *Q. wutaishanica* communities in the two regions. After sampling investigation, we sort out and selected 20 pairs of congeneric common species (20 species on LP and 20 species in QM) and 11 pairs of congeneric endemic species (11 species on LP and 11 species in QM), a total of 31 pairs of congeneric species (62 species in two regions; Figure 1) in six plots across two regions. Because of the interaction between environmental gradients and species, the interspecific differences in traits may offset or strengthen the effects of environmental gradients on traits. Therefore, it is necessary to exclude the influence of genetic background. The control of phylogenetic relatedness is included in the sampling design to exclude the influence of genetic background as much as possible (Fine et al., 2006; Baraloto et al., 2007). In particular, we selected 18 special congeneric species in six genera (*Crataegus*, *Cotoneaster*, *Quercus*, *Euonymus*, *Acer*, and *Lonicera*) from the total 62 species, that is, three species in each genus, including a common species in the two regions and two endemic species limited to LP or QM (Figure 1). We tested the first hypothesis by 18 special congeneric species to accurately exclude the influence of genetic background.

Within each plot, three to five healthy and mature individuals were randomly selected from each target species. Three to five sun-exposed branches with well-developed leaves were collected. The diameter of the base of the cut branches was 6–8 mm, and the length was 15–30 cm. After cutting, branches were immediately sealed in opaque plastic bags, humidified by moist paper towels and transported back to the laboratory for measurements of economic traits and physiological hydraulic traits. Three to five leaves and branches (5–7 cm) were immediately fixed in FAA70 (formaldehyde, acetic acid, and 70% ethanol) for analyses of hydraulic traits (stomatal, veinal traits and anatomical structures; Table 1).

**Table 1:** List of 13 functional traits measured at the branch, leaf, and whole-plant level for this study with corresponding abbreviations and units.

	Plant traits	Abbreviation	Unit	Organ
Hydraulic traits	Height	Height	m	Whole plant
	Wood specific gravity	WSG_branch_	g·cm^−3^	Branch
	Leaf area: sapwood area	Al:As		Branch
	Maximum vessel diameter	Vd_max_	μm	
	Turgor loss point	Ψ_tlp_	MPa	Leaf
	Vein density	VD	mm·mm^−2^	Leaf
	Stomatal density	SD	mm^−2^	Leaf
	Stomatal length	SL	μm	Leaf
Economic traits	Maximum net photosynthetic rate	P_max_	mmol·m^−2^·s^−1^	Leaf
	Leaf dry matter content	LDMC	g·g^−1^	Leaf
	Leaf dry mass per area	LMA	g·m^−2^	Leaf
	Leaf thickness	LT	μm	Leaf
	Leaf tissue density	TD	g cm^−3^	Leaf

### Trait Selection

We measured 13 functional traits (eight hydraulic traits and five economic traits) of 62 species growing in *Q. wutaishanica* forests on LP and in QM (Table 1). Eight hydraulic traits include stomatal density [stomatal density (SD), vein density (VD), wood-specific gravity (WSG_branch_), lower leaf area: sapwood area (Al: As), stomatal length (SL), turgor loss point (Ψ_Tlp_), maximum vessel diameter (Vd_max_) and height (Height)]. Five economic traits include [leaf dry matter content (LDMC), leaf tissue density (TD), leaf dry mass per area (LMA), Leaf thickness (LT) and maximum net photosynthetic rate (P_max_)].

### Economic Traits

We scanned and measured surface areas of each fresh leaf with Motic Images Plus 6.0 software (Motic China group, Xiamen, China). The fresh mass was measured with electronic balance (one ten-thousandth). Thereafter, samples were oven-dried for 72 h at 70°C and weighed as leaf dry mass. Leaf mass per area (LMA, g m^−2^) was calculated as the ratio of dry mass to leaf surface area (Yin et al., 2018). Leaf dry matter content (LDMC, g g^−1^) was calculated as the ratio between leaf dry mass and fresh mass (Zhao et al., 2021). Leaf thickness (LT, μm) was measured through transverse sections using Image-Pro Plus 6.0, avoiding the influence of major veins. Ten to twenty measurements were made for each leaf. Leaf tissue density (TD, g cm^−3^) was calculated as the ratio of LMA to LT (Yin et al., 2018). The photosynthetic rate was determined using the portable photosynthesis system (Li-6,800, Li-Cor, Lincoln, NE, United States) between 9:00 and 11:00 in the field. The temperature was controlled at 20°C–25°C in ambient CO_2_, and the airflow rate was set at 500 μmol s^−1^. The photosynthetically active radiation (PAR) gradients were 1800, 1,500, 1,200, 1,000, 800, 600, 400, 200 and 0 μmol m^−2^ s^−1^. Maximum net photosynthetic rate (P_max_, μmol m^−2^ s^−1^) was obtained by fitting the empirical equation of the least square method adopted by Bassman and Zwier (1991).

### Hydraulic Traits

For vein density assessments, 2 cm^2^ of leaf area were sampled in the central region, immersed in 10% NaOH in an oven at 65°C for 4–12 h. Samples were repeatedly washed with deionized water for about 30 min, immersed and bleached in 10% H_2_O_2_ for 10–30 min and then, washed again in deionized water. Sections were next stained with safranin for 30 min. The sections were then dehydrated using graded ethanol series and immersed in xylene/ethanol absolute (1:1) solution and xylene. Stained sections were mounted, photographed and then, analyzed using Image-Pro Plus 6.0 (Media Cybernetics, United States). The total length of veins per unit area was measured as vein density (VD, mm mm^−2^; Zhao et al., 2021).

Leaf stomatal density (SD, mm^−2^) and stomatal length (SL, μm) were measured on three leaves for one by the nail-polish imprint method (Zhao et al., 2016; Yin et al., 2018). Stomatal prints were observed and photographed under a Classica SK200 digital light microscope (at ×10 magnification; Motic ChinaGroup Co., Ltd., China). More than 20 fields randomly were selected per leaf and a photograph of 200 × 200 μm in the area was selected for analyses using Image-Pro Plus 6.0. The density of stomata (SD, mm^−2^) was calculated by counting all stomata for each specified area (200 × 200 μm) and dividing this number by the area. We measured the length of the guard cells as stomatal length (SL, μm).

We used the paraffin method to get a transverse section of the petiole vessel (Zhao et al., 2021). The paraffin sections were observed and photographed under a light microscope at ×40 magnification equipped with a digital camera. Using Image-Pro Plus 6.0, we assessed the diameter of petiole conduits as described by Schulte (1999) to get the maximum vessel diameter (Vd_max_, μm).

Leaf water potential (Ψ_leaf_, MPa) was measured using a 3,115 portable plant water potential pressure chamber (SEC., Ltd., United States) in consecutive sunny days of mid-July 2019. Leaf samples from three individuals of each species were collected and immediately sealed in opaque plastic bags, humidified by moist paper towels, and transported back to the laboratory. First, leaves were weighed to obtain the initial fresh mass and then placed in a pressure chamber to determine the initial water potential. We measured leaf mass and water potential periodically during slow desiccation of the sample in the natural condition. Samples were oven-dried for 72 h at 70°C and weighed as leaf dry mass. Finally, pressure–volume curves were elaborated according to Tyree and Hammel (1972) to calculate the leaf turgor loss point (Ψ_Tlp_, MPa).

For one individual, 5 cm long branches were cut from the base of the annual branches on the upper part of the species under good light conditions. Then, we removed the bark and determined the wood density (WSG_branch_; Zhao et al., 2021). We used the drainage method to get the volume of the branches, and the dry mass was obtained by weighing scales with an accuracy of 0.0001 g after oven-drying at 70°C for 72 h. The WSG_branch_ is the dry mass of the annual branch divided by the volume of the branch (Zhao et al., 2021). We measured the diameter of the branches by digital calipers (HITEC MESSTECHNIK., Ltd., Germany). Heartwood and pulp were subtracted, and the sapwood area (As) of the branches was obtained. Then, all the leaves on the branches were collected to calculate the total leaf area (Al) and the ratio of total leaf area to sapwood area (Al: As). We estimated the total height of all trees by laser rangefinder. The mean height per species was the average of the five individuals sampled for that species.

### Data Analysis

We used paired *t*-tests (SPSS, Chicago, IL, United States) to evaluate whether 18 special congeneric species (common species and endemic species) in six genera have divergent hydraulic and economic traits. Relationships between economic and hydraulic traits were analyzed with Pearson’s correlation (SPSS, Chicago, IL, United States). Linear regression analyses were used to examine the correlations of traits (SigmaPlot, SPSS Inc., Chicago, IL, United States). Standardized major axis (SMA) estimation (R 3.2.2 statistical platform) was used to determine whether the correlations between economic and hydraulic traits changed with environmental water availability. Multivariate associations of leaf traits were analyzed with a principal component analysis (PCA) in CANOCO software for Windows 4.5 (Microcomputer Power, Ithaca, NY, United States).

## Results

### Congeneric Species Differ in Hydraulic Traits Across Regions

**Figure 2:**
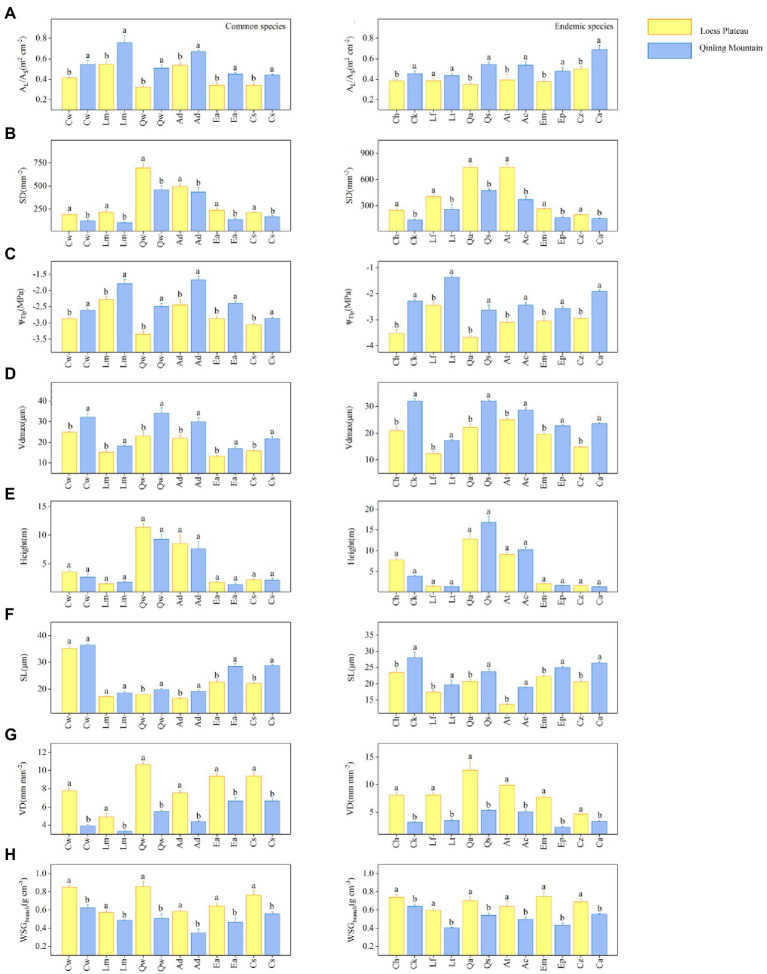
Eight hydraulic traits: **(A)** leaf area: sapwood area (A_l_: A_s_), **(B)** Stomatal density (SD), **(C)** Turgor loss point (Ψ_Tlp_), **(D)** Maximum vessel diameter (Vd_max_), **(E)** Height (height), **(F)** Stomatal length (SL), **(G)** Vein density (VD), and **(H)** Wood specific gravity (WSG_branch_) for 18 special congeneric pairs of species (common species and endemic species in six genera) in Loess plateau (LP; low soil water availability) and Qinling Mountain (high soil water availability). The species abbreviations are shown in Figure 1. Error bars represent 1 SE, and different letters indicate significant differences between regions (*p* < 0.05).

**Figure 3:**
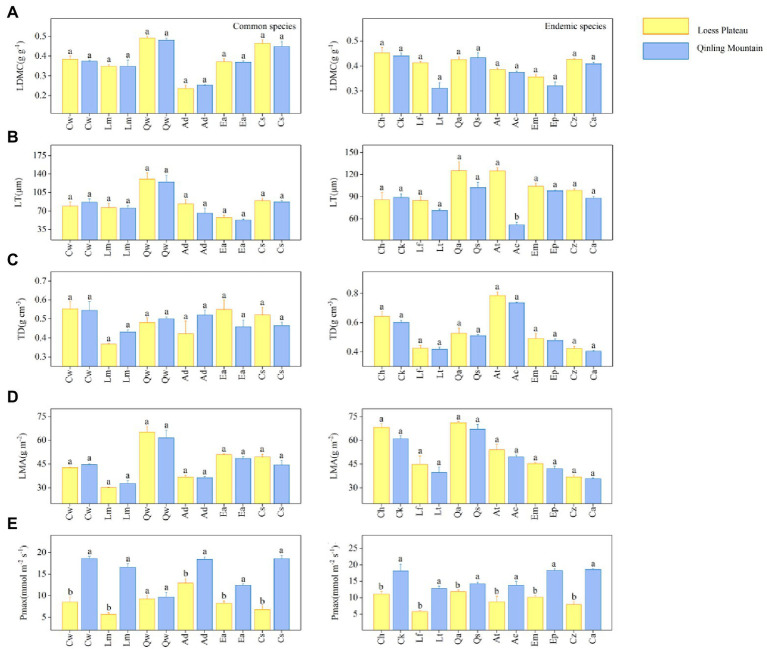
Five economic traits: **(A)** Leaf dry matter content (LDMC), **(B)** Leaf thickness (LT), **(C)** Leaf tissue density (TD), **(D)** Leaf mass per area (LMA), and **(E)** Maximum net photosynthetic rate (P_max_) for 18 special congeneric pairs of species (common species and endemic species in six genera) in Loess plateau (low soil water availability) and Qinling Mountain (high soil water availability). The species abbreviations are shown in Figure 1. Error bars represent 1 SE, and different letters indicate significant differences between regions (*p* < 0.05).

The results showed significant differences in hydraulic traits among 18 special congeneric species in six genera under different soil water availability. Within each genus, common species on LP had lower values of A_l_: A_s_, SL (except for *Crataegus wilsonii*), Vd_max_, and more negative Ψ_Tlp_ values, but higher values of SD, VD and WSG_branch_ values than in QM (Figure 2). For example, the common species of *Quercus* is *Q. wutaishanica*. The SD of *Q. wutaishanica* on LP was higher than that in QM (Figure 2B). There was non-significant difference for Height (Figure 2E). For economic traits, LDMC, LMA, LT, and TD of common species did not differ significantly across regions, except that P_max_ showed significant differences (higher in QM than on LP; Figure 3).

In each of six genera, the congeneric endemic species had similar results with the common species. Except for Height, the other seven hydraulic traits differ significantly across regions (Figure 2). Compared with species in QM, species on LP tended to have lower values of A_l_: A_s_, SL, Vd_max_ and more negative Ψ_Tlp_ values. SD, VD and WSG_branch_ values were higher in QM than on LP. For example, the endemic species of *Quercus* are *Q. acutissima* on LP and *Q. serrata* in QM. The SD of *Q. acutissima* was higher than that of *Q. serrata*. Similarly, most of the five economic traits (LDMC, LMA, LT (except for *Acer* spp.) and TD) showed no significant differences between the two regions, except that the differences of P_max_ were significant (higher in QM than on LP; Figure 3).

### Relationships Between Hydraulic and Economic Traits Differ Across Two Regions

**Figure 4:**
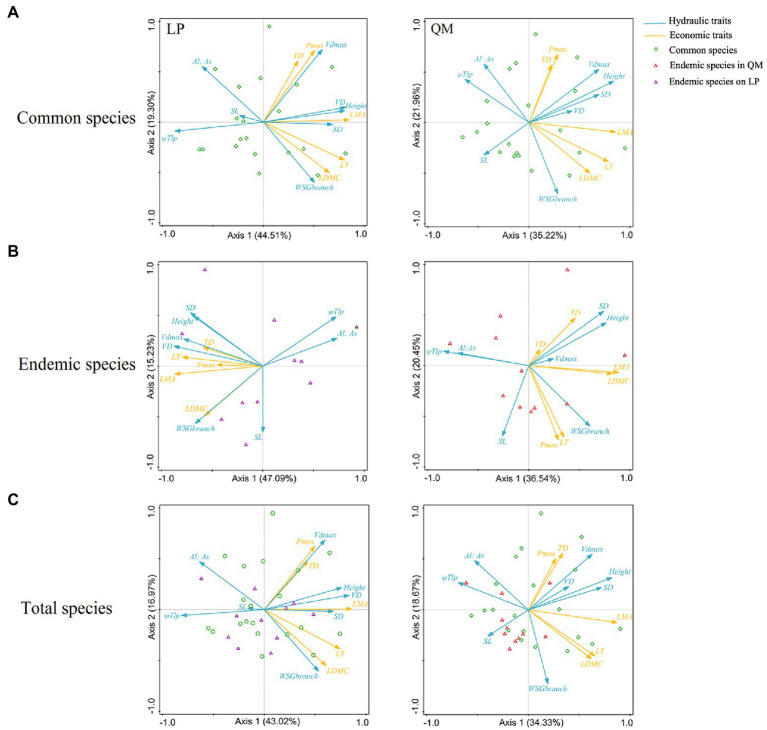
Principal component analysis (PCA) between hydraulic and economic traits on cross-species means among congeneric species **(A)** (20 pairs of congeneric common species, **(B)** 11 pairs of congeneric endemic species and **(C)** 31 pairs of congeneric species). Values in parentheses in the axis labels are percentages explained by the first two components. Eight hydraulic traits were included: SD, stomatal density; SL, stomatal guard cell length; Vd_max_, maximum vessel diameter; Height; Al:As, leaf area: sapwood area ratio in the branch; VD, vein density; WSG_branch_, wood specific gravity of branch; Ψ_Tlp_, turgor loss point. Five economic traits were included: LMA, leaf dry mass per area; LT, leaf thickness; TD, leaf tissue density; LDMC, leaf dry matter content; and P_max_, maximum net photosynthesis rate.

The PCA of 20 common species on LP showed that economic traits could be divided into three groups and were coupled with some hydraulic traits, respectively. P_max_ and TD, coupled with Vd_max_; LDMC and LT, coupled with WSG_branch_ and A_l_: A_s_; LMA, coupled with VD, SD, height, and Ψ_Tlp_. But these were less obvious for 20 common species in QM (Figure 4A). We found similar results in total 31 pairs of species and 11 pairs of endemic species (Figures 4B,C), that is, the relationships between hydraulic and economic traits were closer on LP than in QM.

### Relationships Between Hydraulic and Economic Traits Changed in Common Species

**Figure 5:**
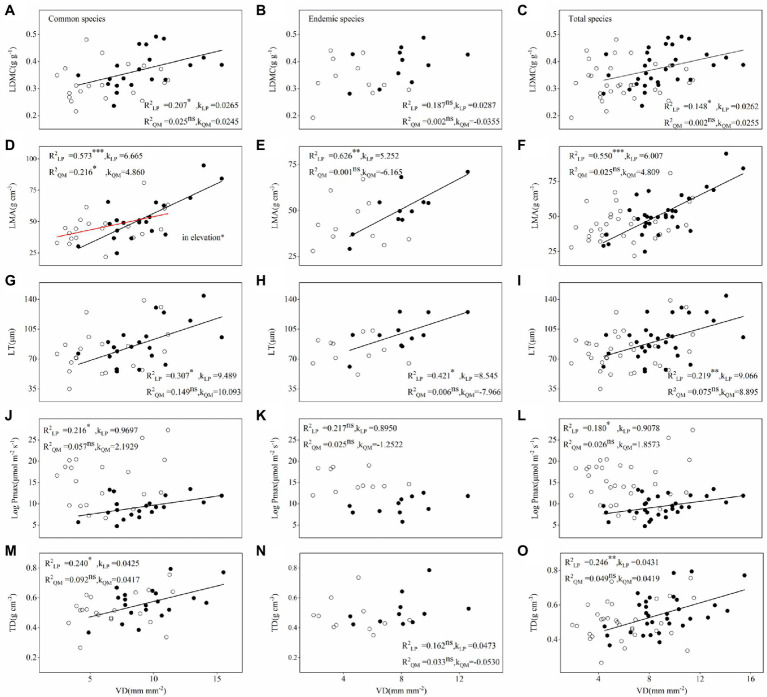
Relationships between VD and **(A-C)** LDMC, **(D-F)** LMA, **(G-I)** LT, **(J-L)** P_max_, **(M-O)** TD of congeneric species (20 pairs of congeneric common species, 11 pairs of congeneric endemic species and 31 pairs of congeneric species) in Loess plateau (low soil water availability; closed circles) and Qinling Mountain (high soil water availability; open circles). Linear regressions were fitted to the data (black regression lines, LP; red regression lines, QM). Values of *R*^2^ are followed by significance level (*, *p* < 0.05; **, *p* < 0.01; ***, and *p* < 0.001). Values of *K* represent slopes of lines.

According to linear regression and SMA analysis, most relationships between hydraulic and economic traits differed between LP and QM, which were closer on LP than in QM for 20 pairs of congeneric common species, 11 pairs of congeneric endemic species and total 31 pairs of congeneric species (Figure 5; Supplementary Figures S2–S6). There was a negative relationship between VD and LMA in common species across regions. LMA decreased with increasing VD, and LMA decreased more on LP than in QM. In addition, some relationships were only found on LP, but not in QM. For instance, the relationships between LDMC and VD were positive on LP, but no relationships were found in QM (Figure 5).

Interestingly, by sorting out the results of linear regression and SMA analysis of all the relationships (Figure 5; Supplementary Figures S2–S6) we found some relationships between hydraulic and economic traits changed (from significant correlation to non-correlation) in common species across regions (from LP to QM), but not in endemic species. For example, Al: As, VD and LDMC; VD and TD; WSG_branch_ and LMA; SD, Ψ_Tlp_, VD, and P_max_ (Table 2).

**Table 2:** According to Figure 5, Supplementary Figures S1–S5, list the relationships between hydraulic and economic traits of 20 pairs of congeneric common species and 11 pairs of congeneric endemic species, which changed with soil water availability [O, significant correlation (*p* < 0.05); \, no correlation (*p* > 0.05)].

Hydraulic-economic	Common species		Endemic species	
	LP	QM	LP	QM
Al: As-LDMC	O	\	\	\
SD-P_max_	O	\	\	\
Ψ_Tlp_-P_max_	O	\	\	\
VD-LDMC	O	\	\	\
VD-P_max_	O	\	\	\
VD-TD	O	\	\	\
WSG_branch_-LMA	O	\	\	\

## Discussion

### The Trade-Off Between Hydraulic Efficiency and Safety in Congeneric Species

Here, we focused on 18 special congeneric species (common species and endemic species) in six genera in *Q. wutaishanica* forests within different soil water availabilities (LP and QM), considering the control of species genetic background. We found significant differences in most hydraulic traits. Across congeneric species, the different combination of hydraulic traits values within each environmental condition showed a significant trade-off between hydraulic efficiency and safety. The result was consistent with our hypothesis that safety vs. efficiency trade-off might affect species co-occurrence at the regional scale. Compared with species in QM, congeneric species on LP tend to adopt conservative water-use strategies and invest in hydraulic traits associated with water transport safety, which indicated a better adaptation to drought environment (Zhao et al., 2021). Both endemic species and common species on LP had higher values of SD, VD, and WSG_branch_, lower Vd_max_, SL, A_l_: A_s_, and more negative Ψ_Tlp_ values, indicating that species on LP invest in higher hydraulic safety at the expense of efficiency, whereas congeneric species in QM had contrasting trait values, suggesting more investment in water transport efficiency. The similar results of endemic species and common species in the same genera fully indicated that differences in hydraulic traits were caused by soil water availability.

Highly embolism resistance is an important feature for species to adapt to low soil water availability (Barigah et al., 2013; Pfautsch et al., 2016; Zhu et al., 2017). Low values of Al: As are associated with hydraulic safety. Because low leaf areas reduce transpiration and ensure water supply, and narrow xylem vessels reduce embolism risk by increasing sapwood area (Togashi et al., 2015; Anderegg and Hillerislambers, 2016; Sande et al., 2019). Also, high values of WSG_branch_ mean thick conduit walls or a great proportion of mechanical tissue, which increase the implosion-resistance (Hacke et al., 2001; Lens et al., 2011; Markesteijn et al., 2011). In low soil water availability, species are favorable to regulate the two traits (WSG_branch_ and A_l_: A_s_) at the branch level to improve the water-resistance (Pratt et al., 2007; Fortunel et al., 2014) and increase hydraulic safety (Cosme et al., 2017). At the leaf level, species can minimize embolism risk by reducing diameters of the maximum vessels in leaf petioles. In low soil water availability, species with narrow vessels appear to be at low risk of air seeding into the water column to ensure water transport safety (Zhao et al., 2021). Increasing leaf vein density is also an effective adaptation to cope with drought (Xiong et al., 2017). The high values of VD increase the paths of water transportation to ensure hydraulic safety (Sack and Scoffoni, 2013). Besides, small and dense stomata (high values of SD and low values of SL) can be more flexible to deal with drought, i.e., close stomata to reduce water loss in time (Franks et al., 2009). Ψ_Tlp_ is also a key factor to determine the tolerance of leaves to drought stress (Bartlett et al., 2012; Maréchaux et al., 2015). It can be used as a proxy of leaf hydraulic vulnerability (Meinzer et al., 2016, 2017; Zhu et al., 2018). Compared with species in QM, species on LP with more negative Ψ_Tlp_ values can maintain positive turgor pressure, certain stomatal conductance, hydraulic conductivity, and photosynthetic gas exchange in low soil water availability (Brodribb and Holbrook, 2003). Therefore, species on LP were more drought resistant. In previous studies, adjustments in Ψ_Tlp_ have also been observed in different water availability (Mitchell and O’Grady, 2015; Farrell et al., 2017; Johnson et al., 2018). There was no significant difference in Height. One possible reason is that species in QM suffer from disturbances caused by winds, heavy rains and animals (Hu et al., 2014; Zhang et al., 2019), which might limit tree growth.

Our results indicated that within-genus variation in hydraulic traits was caused by soil water availability. Different combinations of hydraulic traits for congeneric species (including common species and endemic species) across two regions with different soil water availability supported the trade-off between hydraulic efficiency and safety, which might affect species co-occurrence at the regional scale. The results were different from Zhu et al. (2017), who found that hydraulic efficiency and safety were decoupled in common species. Zhu et al. (2017) mainly discussed that distinctive dimorphic xylem vessels (extremely large vessels and many small vessels) may assure both hydraulic efficiency and safety, which are the reasons of decoupled relationships between hydraulic efficiency and safety in common species. The extremely large vessels allow for high hydraulic efficiency, while the many small vessels allow for high hydraulic safety (Rosell and Olson, 2014). However, many other hydraulic traits can confer hydraulic efficiency or safety in addition to xylem vessels, such as vein density (Sack and Holbrook, 2006; Brodribb et al., 2007). It is obviously not convincing to only considering xylem vessels. No significant differences were found in most economic traits. Our results suggested that hydraulic traits are important in determining the mechanisms of species’ response to drought and may be important for predictions of future species distribution (Anderegg et al., 2012).

### Relationships Were Closer in Drought Region (LP) Than in Humid Region (QM)

Relationships between economic and hydraulic traits were coupled in two regions but were closer couple on LP than in QM. The results were different from previous studies (Li et al., 2015; Zhang et al., 2015; Liu et al., 2020), which showed that hydraulic traits and economic traits were decoupled. The closely relationships of species on LP indicated the principle of optimization in water transportation and CO_2_ assimilation (Wright et al., 2004) and the adaptation mechanism in low water availability (Yin et al., 2018).

We found three groups (TD and P_max_; LT and LDMC; LMA) that were, respectively, coupled to several hydraulic traits on LP, but were not obvious in QM. TD has an important influence on the structure and function of leaves (Kitajima and Poorter, 2010). Under low soil water availability, high values of TD can reduce water loss and increase photosynthetic capacity (Mediavilla et al., 2001; Niinemets, 2001; Elizabeth et al., 2014). Therefore, TD and P_max_ are closely related and coupled with Vd_max_. The diameters of vessels affect water transport capacity (Zhu et al., 2017). According to Hagen–Poiseuille’s law, hydraulic conductance efficiency is proportional to the sum of the vessel diameters to the fourth power (Zimmermann, 1983). High values of LDMC and LT indicated that species have high resource utilization and strong resistance to external water stress and retain water (Garnier et al., 2001). LDMC and LT were coupled with WSG_branch_ and A_l_: A_s_, which indicated that drought-resistant plants improve their hydraulic safety (WSG_branch_ and Al: As, as the discussion in the first part) at the cost of increasing the carbon investment in leaf tissue construction (LDMC and LT; Simonin et al., 2012). LMA is an important leaf carbon economic trait and the core of complex and diversified relational network among leaf economics spectrum (Osnas et al., 2013). On one hand, high values of LMA represent strong wilting resistance and competitiveness against water stress (Baltzer et al., 2008; Rose and Hertel, 2013). VD, Ψ_Tlp_, and stomatal characteristics (such as SD, SL) are also important indexes to measure drought resistance (Martínez-Vilalta et al., 2014). Thus, the coupled relationships between LMA and VD, Ψ_Tlp_, SD, SL confer the extremely strong resistance against hydraulic failure (Blonder et al., 2011; Kardiman and Ræbild, 2017). On the other hand, leaf structural trait (LMA) and leaf hydraulic traits (VD, Ψ TLP, SD, SL) were coupled, indicating that there is a trade-off between the carbon investment allocated to the leaf water transport system and the carbon investment allocated to the construction of leaf structure (Villagra et al., 2013).

Water transport and CO_2_ diffusion are the key processes that determine the assimilation efficiency of terrestrial plants (Flexas et al., 2013). As many studies showed that for plants species the greatest biophysical barrier to survival is the ability to maintain high carbon gain while avoiding desiccation (Nardini and Luglio, 2014). Through the close relationships, species on LP tended to improve photosynthetic carbon assimilation efficiency and the resistance to low soil water availability, then form corresponding morphological structures and strategies (Zhao et al., 2021). Similar results were also found in woody angiosperms on the LP (Yin et al., 2018). By comparison, species in QM with high soil water availability tend to form more flexible combinations in adaption to the environment. Like the relationships between hydraulic efficiency and safety, the results suggested that relationships between hydraulic and economic traits also played a role in constraining species distribution across regions.

### Common Species Changed Relationships Within Different Soil Water Availability

Species distribution is greatly related to water conditions (Cosme et al., 2017). Changes in soil water availability can lead to changes in species composition of communities, which are determined through the process of species migration and substitution (Parmesan and Yohe, 2003; Zhao et al., 2021). Zhu et al. (2017) reported that the reason for common species occurrence in two forests with contrasting soil water conditions was that they could escape the hydraulic trade-off. In our study, there was hydraulic trade-off in common species. Therefore, the causes for common species’ wide occurrence in two forests need further research.

The hydraulic safety and efficiency, water transportation and CO_2_ assimilation trade-offs affect species co-occurrence at the region scale. Species on LP had strong drought-resistant ability and maximize their survival at the expense of hydraulic efficiency and CO_2_ assimilation efficiency. Through sorting out all the relationships between hydraulic and economic traits, we found that seven pairs of relationships (Al: As, VD and LDMC; VD and TD; WSG_branch_ and LMA; SD, Ψ_Tlp_, VD, and P_max_) changed (from significant correlation to non-correlation) in common species across regions (from LP to QM), but not in endemic species. The changes in relationships might be a reason for common species’ wide occurrence in the two *Q. wutaishanica* forests with different water availability and for endemic species only distinctly occurring in either LP or QM.

Common species can be found in two regions, which indicates a stronger ecological adaptability to different water availability than endemic species. We found that common species were sensitive to water availability and capable of changing the hydraulic and economic traits trade-offs in time. On LP with low soil water availability, the significant correlations make common species to adapt to the environment by “increasing resources and reducing expenditure” (Wright et al., 2004; Yin et al., 2018). Whereas in QM with high water availability, non-correlation indicated that common species adapt to the environment by flexible combinations. Endemic species were similar in morphology but were different in ecological and adaptive strategies to occupy the most suitable region in *Q. wutaishanica* forests within different water conditions. On LP with low soil water availability, the development of community is sensitive to water (Xu et al., 2016; Yin et al., 2018). Long-term adaptations to drought allow drought-tolerant species to survive. On the other hand, water stress may also limit species migration and thus accelerate the process of the formation of LP regional endemic species (Parmesan and Yohe, 2003). By contrast, QM with abundant water inhibited the dominance of drought-tolerant species to some extent but met water demand of acquired species and improved their competitiveness to inhibit the development of other species in the community. Thus, endemic species on LP are unable to establish. Therefore, endemic species can only be distributed in a certain region without the change of the relationships between traits.

## Conclusion

This study showed significant differences in hydraulic traits and not in most economic traits of congeneric species across two regions. The different hydraulic traits combinations indicated the trade-off between hydraulic safety and efficiency. We also found the trade-off between hydraulic and economic traits, which were more closely on LP than in QM. The two trade-offs affect species co-occurrence at the regional scale. Seven pairs of relationships changed in common species across regions, but not in endemic species, which may be the reason for differences in the species composition of the same community type. The changes in common species might explain their distributions across regions, but endemic species can only be distributed in a certain region because they lack variation. The results are helpful to understand distributions and adaptive strategies of species, as well as the importance of both hydraulic and economic traits during community assembly.

## Data Availability Statement

The original contributions presented in the study are included in the article/Supplementary Material, further inquiries can be directed to the corresponding author.

## Author Contributions

YZ and MY conceived and designed the experiments and wrote the manuscript. YZ, JZ, JX, YC, PL, JQ, XW, and CL performed the experiments. YZ and JZ analyzed the data. All authors contributed to the article and approved the submitted version.

## Funding

This study was financially supported by the National Science Foundation of China (41871036), the Shaanxi Science and Technology Program (2020JQ-580), China Postdoctoral Science Foundation (2018M643717).

## Conflict of Interest

The authors declare that the research was conducted in the absence of any commercial or financial relationships that could be construed as a potential conflict of interest.

## Publisher’s Note

All claims expressed in this article are solely those of the authors and do not necessarily represent those of their affiliated organizations, or those of the publisher, the editors and the reviewers. Any product that may be evaluated in this article, or claim that may be made by its manufacturer, is not guaranteed or endorsed by the publisher.

## Supplementary Material

The Supplementary Material for this article can be found online at: https://www.frontiersin.org/articles/10.3389/fpls.2022.902509/full#supplementary-material

Supplementary Figure S1 Barplot comparing soil water content (SWC) among per plot between LP and QM. Error bars represent 1 SE, and different letters indicate significant differences between regions (*p* < 0.05).

Supplementary Figure S2 Relationships between Al: As and LDMC, LMA, LT of congeneric species (20 pairs of congeneric common species, 11 pairs of congeneric endemic species and 31 pairs of congeneric species) in Loess plateau (LP; low soil water availability; closed circles) and Qinling Mountain (QM; high soil water availability; open circles). Linear regressions were fitted to the data (black regression lines, LP; red regression lines, QM). Values of R2 are followed by significance level (*, *p* < 0.05; **, *p* < 0.01; and ***, *p* < 0.001). Values of K represent slopes of lines.

Supplementary Figure S3 Relationships between SD and LMA, LT, Pmax.

Supplementary Figure S4 Relationships between ΨTlp and LDMC, LMA, LT, Pmax.

Supplementary Figure S5 Relationships between Vdmax and LMA, TD.

Supplementary Figure S6 Relationships between WSGbranch and LDMC, LMA, LT.
