# Supplementary figures and images for: Effects of Water Availability on the Relationships Between Hydraulic and Economic Traits in the Quercus wutaishanica Forests
Source: Front Plant Sci. 2022 May 26;13:902509. doi: 10.3389/fpls.2022.902509 (PMC9199496; doi:10.3389/fpls.2022.902509)

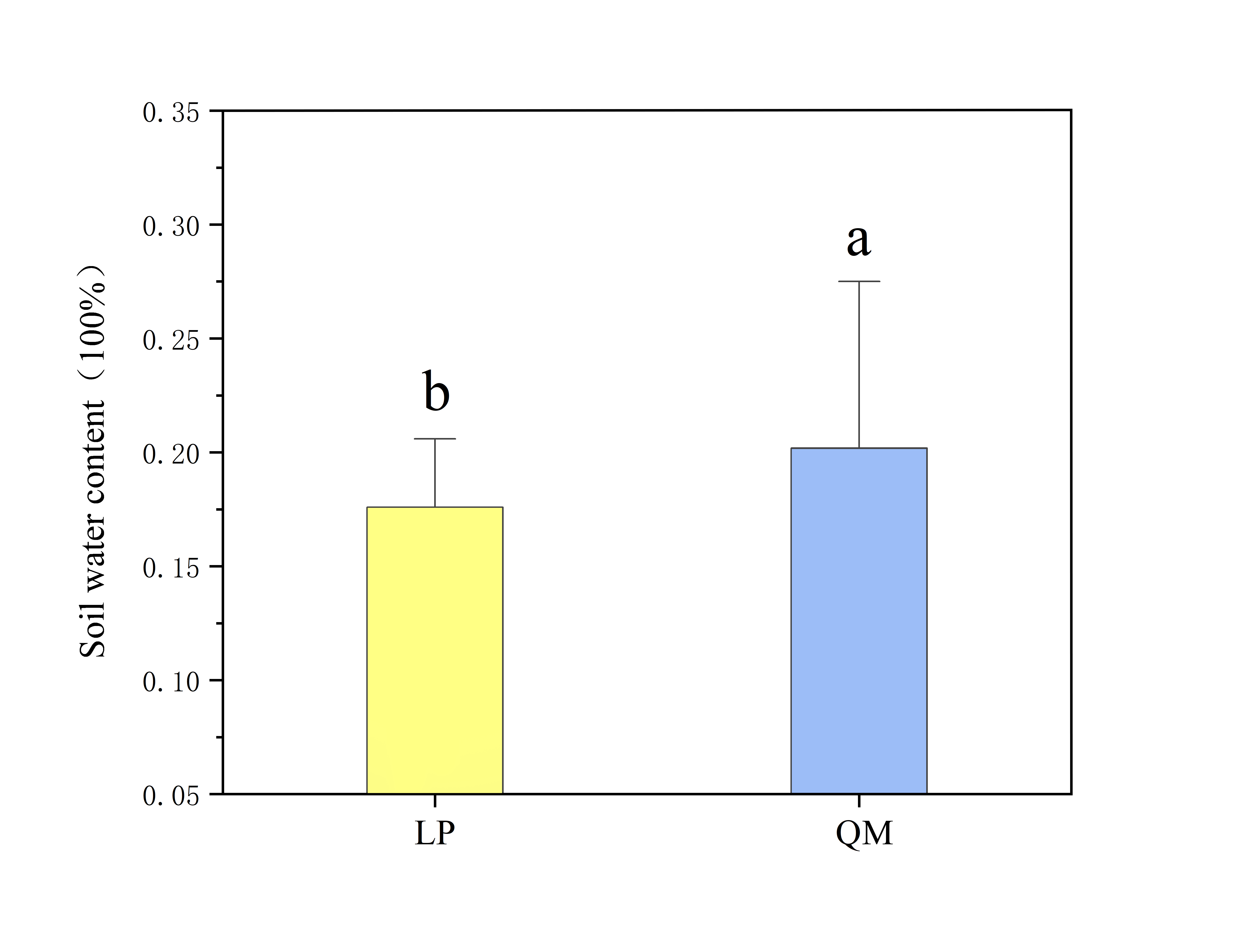

Supplement: Supplementary Figure S1 — Barplot comparing soil water content (SWC) among per plot between LP and QM. Error bars represent 1 SE, and different letters indicate significant differences between regions (p < 0.05). [file Image_1.tif]
