# Supplementary material for: Effects of Water Availability on the Relationships Between Hydraulic and Economic Traits in the Quercus wutaishanica Forests
Source: Front Plant Sci. 2022 May 26;13:902509. doi: 10.3389/fpls.2022.902509 (PMC9199496; doi:10.3389/fpls.2022.902509)
Supplement: Supplementary file 7 [file Data_Sheet_1.PDF]

Table S1 List of the values of the linear regressions. Values of R2 are followed by significance level (*, P < 0.05; **, P < 0.01; ***, P < 0.001). Values of K represent slopes of lines.

**Relationship**	**Loess Plateau**						**Qinling Mountains**					
	Common Species		Endemic Species		Total Species		Common Species		Endemic Species		Total Species	
	R^2	Slope	R^2	Slope	R^2	Slope	R^2	Slope	R^2	Slope	R^2	Slope
Al:As-LDMC	0.418^{**}	-0.7165	0.270^{ns}	-0.7094	0.308^{***}	-0.7026	0.055^{ns}	-0.5193	0.333^{ns}	-0.5187	0.121^{ns}	-0.5159
Al:As-LMA	0.234^{*}	-1.4784	0.584^{**}	-1.1776	0.303^{***}	-1.3510	0.256^{*}	-0.9721	0.560^{**}	-0.8676	0.357^{***}	-0.9254
Al:As-LT	0.309^{*}	-1.224	0.498^{*}	-0.9821	0.254^{***}	-1.1598	0.246^{*}	-1.158	-0.122^{ns}	-0.6594	0.185^{*}	-0.9827
SD-LMA	0.303^{*}	0.5582	0.472^{*}	0.4945	0.342^{***}	0.5404	0.393^{**}	0.4227	0.315^{ns}	0.5035	0.368^{***}	0.4467
SD-LT	0.299^{*}	0.4620	0.549^{*}	0.4124	0.342^{***}	0.4639	0.128^{ns}	0.5036	0.016^{ns}	0.3826	0.091^{ns}	0.4743
SD-P_{max}	0.217^{*}	0.4891	0.001^{ns}	0.4487	0.121^{ns}	0.4795	0.063^{ns}	0.6102	0.012^{ns}	-0.2953	0.128^{ns}	0.5462
Ψ_{Tlp}-LDMC	0.362^{**}	-0.1845	0.489^{*}	-0.1514	0.398^{***}	-0.1728	0.282^{*}	-0.1452	0.255^{ns}	-0.1654	0.393^{**}	-0.1471
Ψ_{Tlp} -LMA	0.434^{**}	-46.39	0.530^{*}	-27.74	0.435^{***}	-39.65	0.277^{*}	-28.79	0.380^{*}	-28.70	0.310^{***}	-27.76
Ψ_{Tlp} -LT	0.312^{*}	-66.03	0.435^{*}	-45.13	0.323^{***}	-59.83	0.207^{*}	-59.80	0.095^{ns}	-37.08	0.158^{*}	-51.31
Ψ_{Tlp} - P_{max}	0.240^{*}	-6.748	0.254^{ns}	-4.727	0.237^{**}	-5.991	0.007^{ns}	-12.992	0.228^{ns}	-5.829	0.020^{ns}	-10.714
VD-LDMC	0.207^{*}	0.0265	0.187^{ns}	0.0287	0.148^{*}	0.0262	0.025^{ns}	0.0245	0.002^{ns}	-0.0355	0.002^{ns}	0.0255
VD-LMA	0.573^{***}	6.665	0.626^{**}	5.252	0.550^{***}	6.007	0.216^{*}	4.860	0.001^{ns}	-6.165	0.025^{ns}	4.809
VD-LT	0.307^{*}	9.489	0.421^{*}	8.545	0.219^{**}	9.066	0.149^{ns}	10.093	0.006^{ns}	-7.966	0.075^{ns}	8.895
VD-Pmax	0.216^{*}	0.9697	0.217^{ns}	0.8950	0.180^{*}	0.9078	0.057^{ns}	2.1929	0.025^{ns}	-1.2522	0.026^{ns}	1.8573
VD-TD	0.240^{*}	0.0425	0.162^{ns}	0.0473	0.246^{**}	0.0431	0.092^{ns}	0.0417	0.033^{ns}	-0.0530	0.049^{ns}	0.0419
Vd_{max}-LMA	0.238^{*}	1.9539	0.451^{*}	3.195	0.248^{**}	2.002	0.288^{*}	0.9867	0.186^{ns}	1.960	0.248^{**}	1.087

Vd_{max} -TD	0.347^{**}	0.0124	0.663^{**}	0.0288	0.389^{***}	0.0144	0.099^{ns}	0.0085	0.230^{ns}	0.0167	0.137^{*}	0.0095
WSG_{branch}-LDMC	0.442^{***}	0.5998	0.606^{**}	0.6458	0.498^{***}	0.6138	0.424^{**}	0.5107	0.033^{ns}	0.7454	0.254^{**}	0.5460
WSG_{branch}-LMA	0.183^{*}	136.71	0.189^{ns}	118.3	0.606^{**}	0.6458	0.025^{ns}	99.43	0.033^{ns}	-129.3	0.033^{ns}	0.7454
WSG_{branch}-LT	0.406^{**}	206.1	0.414^{*}	192.5	0.418^{***}	205.6	0.292^{*}	205.3	0.093^{ns}	167.1	0.209^{*}	186.6
